# Identification of divergent WH2 motifs by HMM-HMM alignments

**DOI:** 10.1186/s13104-015-0981-7

**Published:** 2015-01-24

**Authors:** Clemens Leonard Weiß, Jörg Schultz

**Affiliations:** Department of Bioinformatics, Biozentrum, University Würzburg, Am Hubland, 97074 Würzburg, Germany

**Keywords:** Actin nucleation, Shootin-1, Spire, WH2 domain, HHblits

## Abstract

**Background:**

The actin cytoskeleton is a hallmark of eukaryotic cells. Its regulation as well as its interaction with other proteins is carefully orchestrated by actin interaction domains. One of the key players is the WH2 motif, which enables binding to actin monomers and filaments and is involved in the regulation of actin nucleation. Contrasting conserved domains, the identification of this motif in protein sequences is challenging, as it is short and poorly conserved.

**Findings:**

To identify divergent members, we combined Hidden-Markov-Model (HMM) to HMM alignments with orthology predictions. Thereby, we identified nearly 500 proteins containing so far not annotated WH2 motifs. This included shootin-1, an actin binding protein involved in neuron polarization. Among others, WH2 motifs of ‘proximal to raf’ (ptr)-orthologs, which are described in the literature, but not annotated in genome databases, were identified.

**Conclusion:**

In summary, we increased the number of WH2 motif containing proteins substantially. This identification of candidate regions for actin interaction could steer their experimental characterization. Furthermore, the approach outlined here can easily be adapted to the identification of divergent members of further domain families.

**Electronic supplementary material:**

The online version of this article (doi:10.1186/s13104-015-0981-7) contains supplementary material, which is available to authorized users.

## Findings

Actin is of vital importance for a wide range of cellular processes. Thus both, its conversion from the globular (G-actin) to the filamentous form (F-actin) and its interaction with other proteins has to be finely tuned. Actin interaction domains play key roles in both processes. This includes, among others, the Wasp-homology domain 2 (WH2) motif [1], which not only binds to G-actin and sometimes F-actin, but is also involved in the nucleation of new actin fibres [2]. It exists additionally in proteins as single or repeated modules, and some WH2 repeats appear to promote interactions with F-actin [2,3]. Contrasting archetypal domains, which are structurally conserved, the WH2 motif is intrinsically disordered. Only when interacting with partners such as actin or neighbouring domains in autoinhibited multimodular proteins, WH2 motifs take on a defined structure [3]. Probably as a result of this structural variability, also its sequence is extremely divergent. Furthermore, with an originally reported length of about 35 amino acids [1], WH2 domains are comparably short. These two features make their identification with standard sequence analysis approaches challenging. Possibly as a result of their divergence, WH2 motifs reported in the SMART database [4] range from 12 to 23 AA underlining the difficulties in their identification. Still, delineation of WH2 motifs in a protein would be a considerable benefit as (i) the function of this protein can be considered as actin binding and (ii) experimental characterization of actin interaction can focus on these specific regions.

One of the most sensitive approaches for the identification of divergent homologs is HHblits, which enables the iterative comparison of a single HMM against a database of HMMs [5]. Thus, when searching against standard protein sequence databases, these have be converted into a HMM database. Usually, this is achieved by sequence based clustering using programs like kClust [6]. Here, we suggest a different, more biologically driven approach. Instead of clustering complete sequence databases de novo, we are relying on annotated orthology relationships. We speculated that the addition of noise by combining sequences from different species will highlight conservation patterns which can be picked up by HHblits. Still, by focusing on orthologs, the domain structure of the encoded proteins should be conserved.

All genomes from the ensembl vertebrate, metazoan, plant, protists and fungi databases, respectively, were analyzed [7]. For each data set, orthology information as annotated by Ensembl was extracted. In the case of the vertebrate data set, the pre-calculated alignments of ortholog groups were downloaded. For the other data sets, each ortholog group was aligned using muscle [8]. Subsequently, a HMM was calculated by the hhblitsdb script of the HHSuite package [9]. As queries, WH2 alignments from SMART [4], Pfam [10] and Prosite [11] were downloaded and translated to HMMs. These were used as input for HHblits searches against the ortholog HMMs databases.

For manual evaluation, a database of 12 Drosophila species currently annotated in Ensembl was built. Following the approaches of domain databases like SMART and Pfam, we used this data set to determine the E-value cutoff. As the first true negative hit had an E-value of 3.5 and the last true positive one of 1.7, we set the gathering cutoff to 2. Of the currently annotated eight WH2 motifs in *Drosophila melanogaster* six were identified when combining searches with the three query HMMs. Interestingly, all eight were identified when searching against the metazoan data set which also included the Drosophila data. This indicates that sufficient variation within the orthologous groups is needed for the identification of conserved signals. Not unexpectedly, the identified sequences differed between the three query HMMs. Thus, also the quality of the domain alignment influences search results.

**Figure 1:**
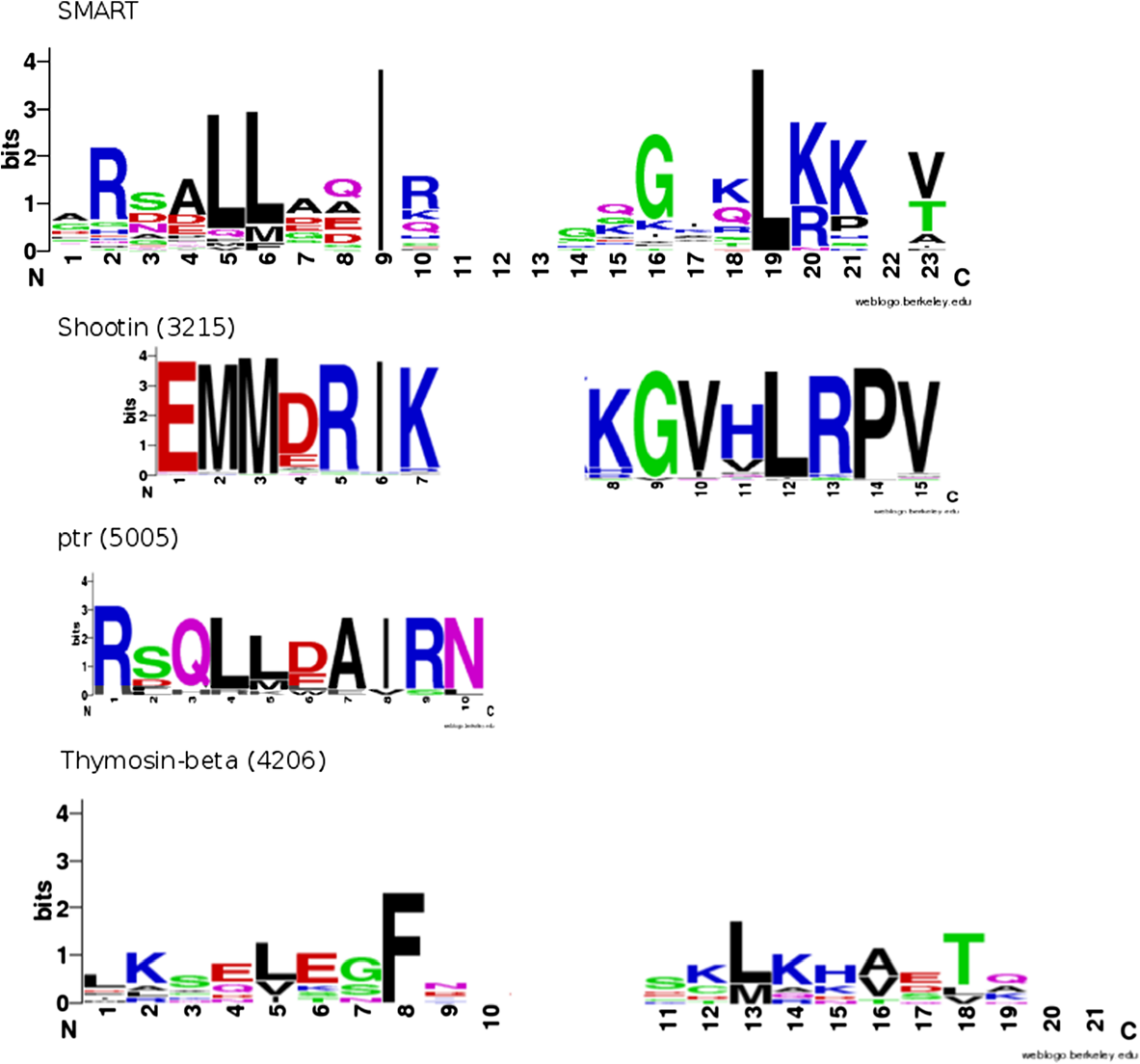
**Sequence Logos of the newly identified WH2 motifs – As a reference, the logo of the SMART WH2 motif alignment is given on top.** Below, sequence logos of the alignments of the shootin-1 and the ptr orthologous WH2 motifs as well as the identified thymosine-β4 sequences are given. Numbers in brackets denote the group identifier used in Additional file 1: Table S1. Sequence logos were generated with WebLogo [12].

Searching against the ensembl genome databases, we identified in total 1,348 WH2 motifs, of which nearly 500 were so far not annotated (Table 1 and Additional file 1: Table S1). This included 61 orthologs of shootin-1 (Group 3215 in Additional file 1: Table S1). To understand why these are not detected by the standard domain databases, we compared the conservation pattern of the shootin-1 WH2 motif with that of the SMART alignment using sequence logos [12] (Figure 1). Indeed, conserved sites harbor amino acids only rarely represented in the SMART alignment. This might be exemplified by the hallmark ‘LL’ motif, which is substituted by ‘MM’ in shootin-1. As this happened on different sites, in the sum the shootin-1 WH2 motif might be just outside of the sequence space covered by the SMART alignment. In rats, this protein is involved in neuron polarization by interaction with actin [13,14]. The identification of a WH2 motif in this protein (ENSRNOP00000061843: from 402 to 417, E-value: 0.00083) predicts the region responsible for the interaction with actin. Similarly, the recently predicted WH2 motif in the *D. melanogaster* protein proximal to raf (ptr) was identified in the according orthologous group (12 members, E-value = 0.17) [15]. Again, a sequence logo representation revealed that the motif is shorter than any WH2 motif represented in the SMART database. This defeats its detection as SMART relies on global alignments. In total, we identified WH2 motifs in 52 ortholog groups comprising 476 sequences for which no WH2 motif was annotated so far.

**Table 1:** **Summary of identified WH2 domains (E-value cutoff 2/0.05)**

	**Drosophila**	**Fungi**	**Metazoa**	**Protozoa**	**Vertebra**	**All**
Annotated found	71/71	18/18	49/48	8/8	735/695	872/831
Annotated not found	23/23	5/5	51/52	12/12	221/260	300/339
New	28/1	3/0	13/1	0/0	433/417	476/419

In further ortholog groups WH2 motifs were not consistently predicted by Ensembl. For these, the WH2 motif is now reliably annotated for all members spanning the according position. A typical example is Spire [16]. So far, its WH2 motif was annotated only for a few species including *D. melanogaster*. We now identified the motif also in the vertebrate proteins including mouse, rat and human. Lastly, we identified a significant similarity to thymosine-β4 domain, which has a WH2 motif like structure despite the very poorly conserved sequence. Indeed, it is mainly the C-terminus which displays commonly conserved positions, whereas the N-terminus varies strongly (Figure 1). Still, the identification of a significant similarity corroborates a suggested common evolutionary origin [17].

In summary, our approach was able to identify a substantial number of WH2 motifs so far overlooked in standard genome databases. Admittedly, this is influenced by the chosen E-value cutoff. Still, we think that this is a valid approach, which is also implemented in Pfam as gathering threshold and in SMART as domain specific cutoff [18]. Differences between the analyzed data sets indicate that the degree of conservation versus variation is of importance for the sensitivity of the approach. Thus one might have to adopt the considered species depending on the domain. In general, the advantage of relying on orthologs instead of de-novo clustering is that the sets are based on a biological assumption - common origin based on speciation - and therefore, the domain content should be conserved. In sequence clustering, this will be only achievable by strict length cutoffs, which might influence the results of clustering. Thus, the approach presented here could be easily adapted for the identification of divergent family members of further domains.

## Additional file

Additional file 1: Table S1. List of all proteins containing newly identified WH2 domains including their sequences.
